# Abnormal B-Cell Maturation and Increased Transitional B Cells in CBL Syndrome

**DOI:** 10.3389/fped.2022.935951

**Published:** 2022-07-28

**Authors:** Francesco Saettini, Tiziana Angela Coliva, Francesca Vendemini, Marta Galbiati, Cristina Bugarin, Riccardo Masetti, Daniele Moratto, Marco Chiarini, Fabiola Guerra, Maria Iascone, Raffaele Badolato, Giovanni Cazzaniga, Charlotte Niemeyer, Christian Flotho, Andrea Biondi

**Affiliations:** ^1^Department of Pediatric Hematology, Fondazione Monza e Brianza per il Bambino e la sua Mamma (MBBM), University of Milano Bicocca, Monza, Italy; ^2^Centro Ricerca Tettamanti, University of Milano Bicocca, Monza, Italy; ^3^Pediatric Hematology-Oncology Unit, Department of Medical and Surgical Sciences (DIMEC), University of Bologna, Bologna, Italy; ^4^Flow Cytometry, Clinical Chemistry Laboratory, Brescia, Italy; ^5^Laboratorio di Genetica Medica, Azienda Socio Sanitaria Territoriale (ASST) Papa Giovanni XXIII, Bergamo, Italy; ^6^Department of Clinical and Experimental Sciences, Pediatrics Clinic and Institute for Molecular Medicine A. Nocivelli, University of Brescia and ASST-Spedali Civili of Brescia, Brescia, Italy; ^7^Department of Pediatrics and Adolescent Medicine, University Children’s Hospital, University of Freiburg, Freiburg, Germany; ^8^Pediatric Department and Centro Tettamanti-European Reference Network PaedCan, EuroBloodNet, MetabERN-University of Milano-Bicocca-Fondazione Monza e Brianza per il Bambino e la sua Mamma (MBBM)-Ospedale, San Gerardo, Monza, Italy

**Keywords:** CBL, RASopathies, RALD, BENTA, ALPS, *CARD11*, splenomegaly, lymphocytosis

## Abstract

CBL syndrome is a Noonan-like RASopathy with heterogeneous clinical phenotype and predisposition to juvenile myelomonocytic leukemia (JMML). Here we describe two patients with identical germline *CBL* mutation and clinical and immune-hematological overlapping features with autoimmune lymphoproliferative syndrome (ALPS) and B-cell expansion with NF-κB and T-cell anergy (BENTA) syndrome. Increased immature/transitional B cells can be depicted in CBL syndrome, ALPS, and BENTA. Nonetheless, our patients here described showed peculiar B-cell phenotype due to increased immature/transitional CD34^+^ B cells. This feature differentiates CBL syndrome from BENTA, pointing toward an abnormal proliferation of B-cell early precursors.

## Introduction

Germline mutations of the *CBL* gene cause CBL syndrome characterized by variable phenotype involving high frequency of neurologic features, vasculitis, mild Noonan syndrome-like features, and predisposition to juvenile myelomonocytic leukemia (JMML) (1). Indeed, more than 90% of patients with JMML harbor mutations in RAS genes (*NRAS* or *KRAS*) or RAS pathway regulators (*PTPN11*, *NF1*, or *CBL*), which collectively share constitutive activation of RAS/MAPK signaling and therefore also called RASopathies (2). *KRAS* and *NRAS* somatic mutations have been identified in RAS-associated autoimmune leuko-proliferative disorders (RALD), which is associated with overexpansion of lymphocytes with hepatosplenomegaly, lymphadenopathy, and autoimmune phenomena, sharing some clinical and hematopathological features with autoimmune lymphoproliferative syndrome (ALPS) (3).

Although the phenotype of children affected by CBL syndrome has been increasingly delineated over time (4, 5), the immunological features of CBL syndrome have not been extensively described. Here we describe two patients with identical germline *CBL* mutation [c.1259G > A; (pR420Q)], expanding the immunological phenotypic spectrum of RASopathies and CBL syndrome. Overlapping features with B-cell expansion with NF-κB and T-cell anergy (BENTA) syndrome and RALD are discussed.

An 8-month-old girl with a previous history of severe infections and leukocytosis was referred to our clinic (P1). Family history was unremarkable. At the age of 4 months, she had pneumococcal encephalitis, sepsis, and status epilepticus with subsequent labyrinthitis ossificans. Abdominal ultrasound showed splenomegaly (spleen diameter of 10 cm) and hydronephrosis in the right kidney. In the following months, several urinary tract infections required two admissions and intravenous antibiotics. She presented with speech and psychomotor delay. Right sensorineural hearing loss was demonstrated.

**FIGURE 1:**
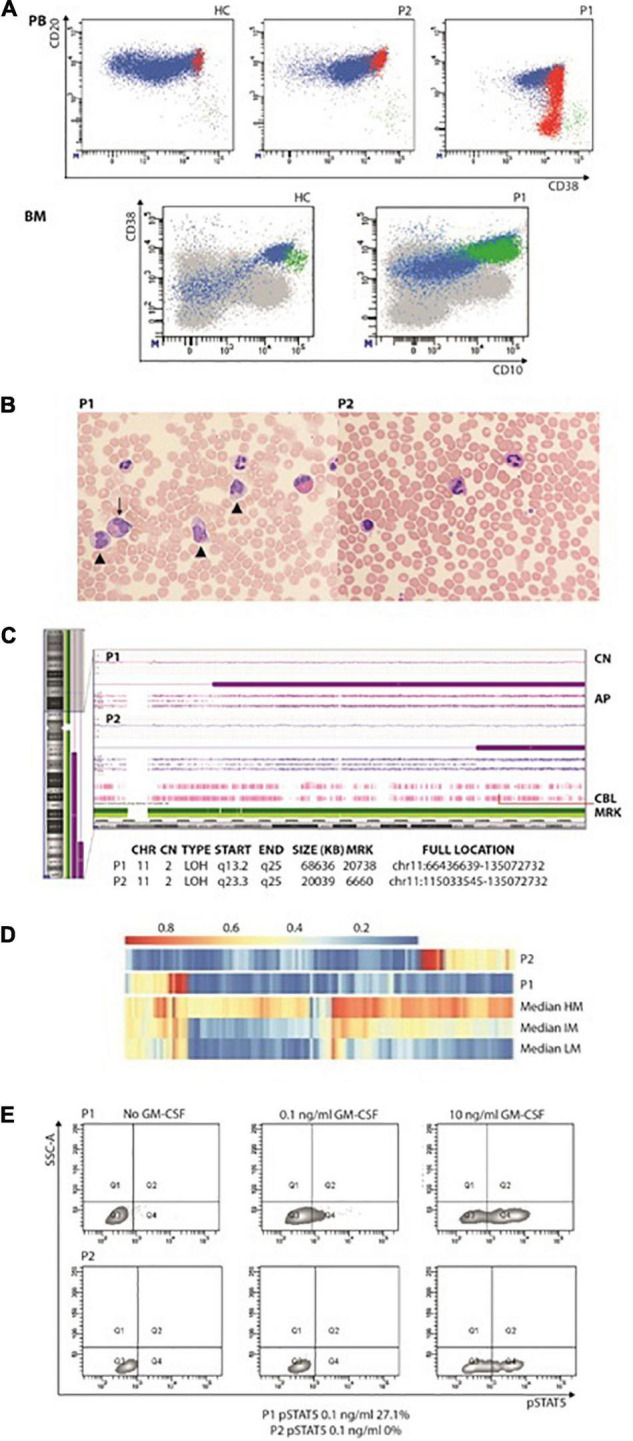
**(A)** Increased immature/transitional B in the peripheral blood (upper panel; in red are represented CD10^+^CD21^–^CD27^–^ transitional or immature B cells, in green CD10^–^CD21^–^CD27^+^ terminally differentiated B cells) and bone marrow (lower panel; in green CD34^+^CD38^+^CD10^+^). **(B)** Peripheral blood smears showing JMML in P1 (left panel): leukocytosis with monocytosis (triangle) and myeloid precursors (arrows). In the right panel, anisocytosis of platelets without leukocytosis or monocytosis in P2’s peripheral blood smear. **(C)** CytoScan HD Array (SNP) analysis: allelic peak (AP) graph showing large copy neutral (CN = 2) loss of heterozygosity (LOH) regions in long arm of chromosome 11, in both patients; common LOH region includes CBL gene at band 11q23.3 (red line). **(D)** DNA methylation patterns in the reported patients showing low-methylation status. **(E)** STAT5 phosphorylation consistent with JMML in P1. AP, allele peaks; CHR, chromosome; CN, copy number state; HC, healthy control; IM, intermediate methylation; HM, high methylation; LM, low methylation; LOH, loss of heterozygosity; MRK, markers/markers number; P1 and P2, patients, START/END, cytoband start/end.

Peripheral blood (PB) tests showed monocytosis (range 1.6–3.69 × 10^9^/l) and thrombocytopenia. B-cell lymphocytosis (range 3.6–5.5 × 10^9^/l) was persistent. B-cell subsets showed a predominant immature/transitional phenotype (Table 1 and Figure 1A). A bone marrow (BM) aspiration showed increased immature B cells (CD10^++^CD19^+^ CD20^+^CD38^++^; Figure 1B). No dysplastic features were noticed and megakaryocytes were rare. Karyotype was normal. Most of these immature B cells still expressed the CD34 antigen either in PB or BM.

**TABLE 1:** Patient characteristics at the time of first evaluation.

	Pt1	Age-matched normal values	Pt2	Age-matched normal values
Age, years	0.5		16	
Hemoglobin, g/dl	12.1	11.5–13.5	13.2	12.0–16.0
Mean corpuscolar volume, fl	86.9	75–87	83.5	78–102
White blood cells, 10^9^/l	23.61	5.2–11.0	8.48	4.4–8.1
Neutrophils10^9^/l	9.43	>1.5	6.57	>1.5
Lymphocytes10^9^/l	9.82	3.4–9.0	1.23	1.4–3.3
Monocytes10^9^/l	3.69	<1.0	0.58	<1.0
Eosinophils10^9^/l	0.51	<0.5	0.07	<0.5
Basophils10^9^/l	0.17	<0.1	0.02	<0.1
Platelets10^9^/l	111	>140	156	>140
HbF,%	1.1	3–15	0.9	0.1–1.2
IgG, mg/dl	622	351–919	1431	604–1909
IgA, mg/dl	29	10–85	172	61–301
IgM, mg/dl	49	38–204	119	59–297
IgEkU/l	956	<33	84	<33
CD3 + 10^9^/l	3.3	1.9–5.9	809	0.72–2.56
CD4 + 10^9^/l	2.77	1.4–4.3	546	0.27–1.88
HLADR + , %	3.9	0.8–6.1	2.3	1.6–12.2
Naïve CD45RA + CCR7 + , %	78.8	68.8–91.7	51.3%	20.4–63.6
RTE CD45RA + CCR7 + CD31 + , %	67.7	42.0–79.0	42.3	11.4–48.1
Centr. mem. CD45RA-CCR7 + , %	14.9	5.6–24.2	30.4	18.7–46.2
Eff mem CD45RA- CCR7-, %	5.0	1.5–8.3	17.0	7.1–38.0
Term diff CD45RA + CCR7 -, %	0.9	0.3–5.9	1.2	0.3–9.1
CD8 + 10^9^/l	0.27	0.5–1.7	0.16	0.18–0.78
HLADR + , %	3.4	1.6–30.2	2.2	2.7–31.7
Naïve CD45RA + CCR7 + , %	78.6	37.9–90.7	39.7	13.1–66.5
Centr. Mem. CD45RA- CCR7 + , %	3.9	2.0–13.0	5.0	2.6–24.5
Eff Mem CD45RA- CCR7-, %	6.3	1.3–27.2	40.0	10.1–47.4
Term Diff CD45RA + CCR7-, %	11.3	2.1–36.1	15.3	5.2–63.5
CD19 + 10^9^/l	4.96	0.61–2.6	0.15	0.09–0.65
RBE CD38 + + CD10 + , %	76.6	16.5–56.5	33.8	2.1–26.1
Naïve IgD + IgM + CD27-, %	17.3	32.2–66.9	50.5	33.7–74.0
CD19 + + CD21low, %	0.7	0.7–6.2	1.2	1.4–13.6
Sw Mem IgD-IgM-CD27 + , %	0.32	0.12–2.3	6.4	2.8–23.4
IgM Mem IgD + IgM + CD27 + , %	1.6	1.6–8.8	7.3	5.1–25.5
Term Diff CD38++ CD27 + CD20-, %	0.42	0.2–8.5	0.7	0.2–8.1
CD3-CD16 + CD56 + 10^9^/l	1.18	0.16–0.95	0.18	0.04–0.74

At 3.5 years, JMML was diagnosed based on complete blood count (CBC) with differential, PB, smear findings and detection of CBL variant (6, 7). The CBC showed persistent monocytosis and thrombocytopenia while PB smear myelocytes and metamyelocytes were detected along with anisocytosis in either erythrocytes or platelets, some of whose were giant (Figure 1C). She has now been followed up for 46 months: over time, thrombocytopenia resolved with the persistence of monocytosis and splenomegaly.

A 16-year-old girl with a previously unremarkable medical history was referred to the Pediatric Hematology outpatient Clinic after splenomegaly was noticed during a routine examination (P2). The patient was born to non-consanguineous parents after an uncomplicated pregnancy. The CBC was normal but the PB smear analysis revealed platelet anisocytosis with giant forms and partially hypergranulated granulocytes were detected (Figure 1C). Lymphocyte subsets showed normal values. A BM aspiration showed dysplastic features. Karyotype was normal. The patient is currently 17 years old and in good clinical condition but with splenomegaly.

Both patients showed normal IgG levels. P1 showed decreased IgA and increased IgE, with no sign of atopy. P1’s and P2’s extensive autoimmunity workup was negative. Both patients had normal level of fetal hemoglobin (HbF).

Severe cytopenia and massive splenomegaly were never observed in both patients; therefore, they were strictly followed up and no further treatment options (6-mercaptopurine and/or low-dose cytarabine or splenectomy) were considered (6).

In both patients’ PBMC, the homozygous variant c.1259G > A;[p.Arg420Gln] located in the exon 9 of *CBL* was detected. P1 was investigated by means of target next generation sequencing (NGS) panels, which comprehended primary immunodeficiency defects (including *CARD11*), while trio whole-exome sequencing (WES) was performed in P2. The Sanger sequencing in DNA extracted from hair follicles detected the variant at the heterozygous status, indicating the germline origin in both patients. The single nucleotide polymorphism (SNP) array performed on PBMC suggested that *CBL* mutations were related to the loss of heterozygosity of chromosome 11q that included the *CBL* gene (Figure 1D). The DNA methylation profile was predicted to fit the low methylation class (Figure 1B). P1 showed p-STAT5 hyper-responsiveness to low doses (0.1 ng/ml) of GM-CSF assayed in CD33^+^CD34^+^ cells (Figure 1E). Due to the identification of CBL mutation, both patients underwent brain magnetic resonance and abdominal Doppler ultrasound without evidence of vasculopathy.

CBL syndrome is a Noonan-like RASopathy with heterogeneous clinical phenotype. Confirming the variant in hair follicles or fibroblasts is crucial in order to prove the germline origin of the genetic lesion and distinguishing Noonan-like syndromes from RALD. To date, more than 50 cases of CBL syndrome have been reported and the majority of them developed JMML (4). JMML is a myeloproliferative/myelodysplastic neoplasm of early childhood characterized by rapidly progressive disease requiring allogeneic hematopoietic stem cell transplantation as potentially curative treatment in the majority of the patients. Older age, elevated HbF levels, and thrombocytopenia at diagnosis correlate with the poor clinical outcome (6). Other main prognostic factors are represented by genetic subtype defined by RAS pathway mutations and methylation status (8). CBL-mutated JMML can follow an aggressive clinical course or evolve to a spontaneous regression of myeloproliferation with persistence of clonal hematopoiesis (1).

Persistent monocytosis, B-cell lymphocytosis with increased transitional B cells, and splenomegaly have been described in patients affected by RALD (3, 9). BENTA syndrome due to germline gain of function *CARD11* mutations is characterized by congenital lymphoid hyperplasia (particularly splenomegaly) driven by excessive, polyclonal accumulation of B lymphocytes (9). B-cell lymphocytosis was constantly present in P1, resembling the picture already reported in RALD, BENTA, and JMML patients (9). Increased transitional B cells in the PB were detected in both of our patients with T-cell numbers within normal pediatric ranges. Our patients and another patient with CBL reported by Tejwani displayed B subset abnormalities (increased CD10^+^ immature/transitional B cells) (10).

Moreover, some differences between BENTA, RALD, and CBL syndrome can be drawn. It has been proposed that in BENTA syndrome, B cells may accumulate from increased B-cell output from the BM, as indicated by elevated CD10^+^ transitional B cells in the PB and consistent with increased immature B cells in the BM (11). Here we have further characterized the B-cell phenotype showing that a consistent number of increased immature/transitional B cells still show the presence of the CD34 antigen (Figure 1B), thus pointing toward an abnormal proliferation of B-cell early precursors rather than increased B-cell output from the BM. Recurrent and/or severe infections are common in BENTA syndrome. Although B-cell lymphocytosis has been linked to chronic EBV infection in patients with BENTA (11), P1 did not encounter EBV. CBL patients do not show decreased memory and class-switched B-cell numbers nor hypogammaglobulinemia.

Overall, these cases expand the phenotypic spectrum of CBL syndrome, which overlaps with RALD and BENTA syndrome due to the increased immature/transitional B cells.

## Data Availability Statement

The raw data supporting the conclusions of this article will be made available by the authors, without undue reservation.

## Ethics Statement

The studies involving human participants were reviewed and approved by the Fondazione MBBM, Monza, Italy. Written informed consent to participate in this study was provided by the participants’ legal guardian/next of kin.

## Author Contributions

FS and FV contributed to the conception and design of the study. FS wrote the first draft of the manuscript. All authors contributed to the article and approved the submitted version.

## Conflict of Interest

The authors declare that the research was conducted in the absence of any commercial or financial relationships that could be construed as a potential conflict of interest.

## Publisher’s Note

All claims expressed in this article are solely those of the authors and do not necessarily represent those of their affiliated organizations, or those of the publisher, the editors and the reviewers. Any product that may be evaluated in this article, or claim that may be made by its manufacturer, is not guaranteed or endorsed by the publisher.
